# Cancer in children and adolescents in the Brazilian Amazon: a population-based study

**DOI:** 10.3332/ecancer.2025.1945

**Published:** 2025-07-15

**Authors:** Isabella Pereira Gadelha, Fernanda Cristina Rosa Alves, Samara Machado Castilho, George Pinheiro Carvalho, Luciana Ferreira dos Santos, Lucrecia Aline Cabral Formigosa

**Affiliations:** 1 Belém Population-Based Cancer Registry, Belém 66093-677, Brazil; 2 State University of Pará, Belém 66065-362, Brazil

**Keywords:** neoplasms, epidemiology, incidence, mortality, child, adolescent

## Abstract

**Background:**

Every year, about 400,000 children are diagnosed with cancer around the world, 90% of them in developing countries. In Brazil, it is estimated that 7,900 cases are expected by 2023, making cancer the second leading cause of death in this age group. Therefore, this study is relevant for strengthening health surveillance and developing strategies to combat childhood and adolescent malignant neoplasms.

**Objective:**

To describe the profile of cancer incidence and mortality in the child and adolescent population of Belém and Ananindeua between 2010 and 2019.

**Methods:**

This is a descriptive study, with a quantitative approach, using data obtained from the Population-Based Cancer Registry (RCBP), referring to cancer incidence and mortality in individuals aged 0–19 residing in Belém and Ananindeua. The following variables were analysed: age, sex, race, morphology and case outcome. The data were organised in Microsoft Excel, using the formula: incidence = (number of new cases/total population) × 100,000. The analyses were descriptive, with emphasis on absolute and relative frequencies.

**Results:**

A total of 846 new cases of malignant neoplasms were registered among the population aged 0–19. The most frequent morphology was leukaemia, with 42.31 cases per 100,000 inhabitants, followed by malignant tumours of the central nervous system, with 17.92, lymphomas, with 15.82 and bone tumours, with 12.27. The incidence is higher among males, with a large number of cases occurring in the age group 15–19. As for the outcome, 66.1% had no information on the patient’s vital status, 29.6% had a confirmed death and only 4.4% had a vital status of alive.

**Conclusion:**

The epidemiological data on childhood and adolescent malignant neoplasms in Belém and Ananindeua was detailed, providing more information on the subject. However, it was difficult to obtain some information from the RCBP system.

## Background

Cancer is one of the world’s main public health problems, due to its high incidence and mortality rates, as well as its social and economic impact. In this context, according to the José Alencar Gomes da Silva National Cancer Institute (INCA, as per its Portuguese acronym), the main neoplasms responsible for affecting the population of children and adolescents (0–19 years old) are leukaemia, central nervous system (CNS) tumours and lymphomas. Thus, surveillance of the disease is extremely important for planning policies to combat, prevent and evaluate the pathology in this population [1].

Every year, about 400,000 children are diagnosed with cancer around the world, 90% of whom reside in developing countries. The most common type of malignant neoplasm varies according to region. However, acute lymphoblastic leukaemia is the most common. In addition, survival in low- and middle-income countries is between 15% and 45%, as opposed to high-income countries, which have a survival rate of around 80% [2].

In Brazil, the estimate for the year 2023 is around 700,000 new cases of cancer, of which 7,900 are related to the public consisting of children and adolescents, with males being the most prevalent, both globally and in Brazil. In addition, cancer is the second leading cause of death among children and adolescents in Brazil [1, 3].

Furthermore, cancer mortality rates are higher in developing countries. In Brazil, the indexes have been stable over the years, but there are regional variations, with the Northern region between 2011 and 2021 having the highest mortality rates in the age groups from 0 to 4 years (26.15%) and from 5 to 9 years (22.97%). This is due to Brazil’s territorial extension, cultural and socioeconomic differences, which make it difficult to access diagnosis and treatment in certain regions [4, 5].

Neoplasms in children and adolescents have peculiarities compared to adults, since lifestyle and behaviours, such as diet, physical activity and unhealthy habits, cannot be considered risk factors for tumour development. In turn, genetic alterations are more relevant to explain the onset of cancer in children [3, 6].

In this context, one of the main problems is the difficulty in making an early diagnosis and starting antineoplastic treatment, which influences the patient’s cure rate and survival. According to Law 12.732/2012, in Brazil, treatment must start within 60 days of diagnosis; however, patients aged 10–19 tend to start treatment within a longer period when compared to patients aged 0–9 [6, 7].

Thus, this study is justified by the crucial role of research on cancer incidence and mortality in strengthening health surveillance systems and informing evidence-based strategies for the prevention, early detection and treatment of childhood and adolescent malignant neoplasms. Additionally, it contributes to one of the World Health Organisation’s Millennium Development Goals: reducing child mortality. Studies indicate that childhood cancer mortality remains high in regions with socioeconomic disparities and limited access to specialised treatment. Northern Brazil faces considerable challenges in ensuring early diagnosis and effective oncological care, underscoring the need for region-specific epidemiological data [4, 8, 9].

Therefore, the objective of this study is to describe the profile of cancer incidence and mortality in the child and adolescent population of Belém and Ananindeua, from 2010 to 2019, based on the data contained in the Population-Based Cancer Registry (RCBP) of Belém do Pará.

## Methods

This is a descriptive study, with a quantitative approach. The data used refers to cancer incidence and mortality for all types of malignant tumours in individuals aged 19 or under residing in the cities of Belém and Ananindeua, obtained from the (RCBP, as per its Portuguese acronym) available at the following website: https://www.inca.gov.br/BasePopIncidencias/Home.action.

The RCBP has information on the incidence of cancer cases and their distribution in a given region within its coverage area. These data are obtained through a process of continuous and standardised collection, with an active search for new cases of cancer. Their variables include: date of diagnosis of the primary tumour, means of diagnosis, extent of the disease, topography, morphology, sex, age group, race, address, occupation and vital status, which are essential for analysing the most frequent type of cancer, mortality rate, incidence, prevalence, diagnosis in relation to sex and specific professions that pose risks to workers [10–12].

The coding of notifications is based on the International Classification of Diseases for Oncology – 3rd Edition (ICD-O 3) for adult records, the International Classification of Childhood Cancer (CICI-3) and the TNM (Classification of Malignant Tumours – 7th Edition) [11].

The inclusion criteria were: cases of malignant neoplasms in individuals aged 0–19 years, 11 months and 29 days, reported between 2010 and 2019. The exclusion criteria were: cases registered with incomplete or missing information regarding the topography and morphology of the malignant neoplasm.

The search in the system was conducted between 02/16/2024 and 06/06/2024 and referred to the historical base from January 2010 to December 2019, in the cities of Belém and Ananindeua in the state of Pará. The following variables were analysed: age, sex, race, tumour morphology and case outcome.

The data were tabulated and organised in Microsoft Excel software, where calculations were made to determine cancer incidence rates. Incidence was calculated using the formula: incidence = (number of new cases/total population) × 100,000, considering the population aged 0–19 residing in Belém and Ananindeua, based on data from the 2010 Census, which resulted in a total of 619,219 inhabitants. The analyses were conducted descriptively, using absolute and relative frequencies to characterise the studied variables [13].

This study used publicly available secondary data extracted from the RCBP. As these data are freely accessible, it was not necessary to submit them to the Research Ethics Committee. The authors declare that they are aware of Resolution nº 466/2012 of the Brazilian National Health Council.

## Results

This study found that 846 new cases of malignant neoplasms were reported by the RCBP in the municipalities of Belém and Ananindeua, in the state of Pará, between 2010 and 2019, in both sexes.

The most common morphology was leukaemia, with 42.31 cases per 100,000 inhabitants, followed by malignant tumours of the CNS, with 17.92 cases per 100,000 inhabitants. Lymphomas appear as the third most common morphological type, with 15.82 cases per 100,000 inhabitants and bone tumours, with 12.27 cases per 100,000 inhabitants.

**Figure 1.**
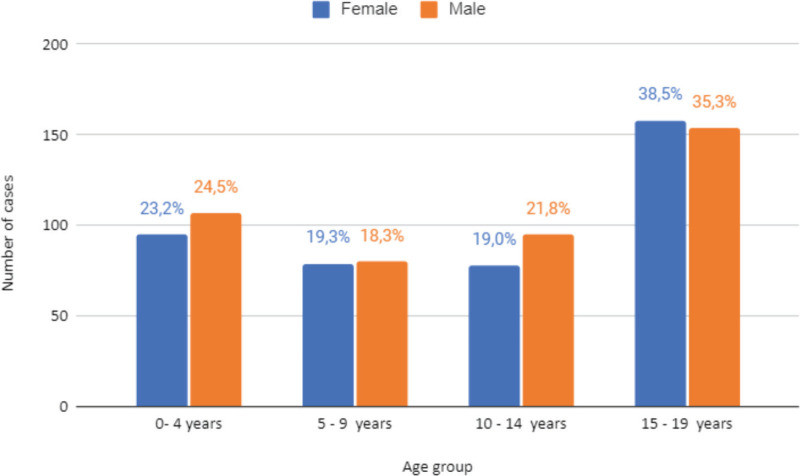
Cases of malignant neoplasms in Belém and Ananindeua between 2010 and 2019, according to sex and age. Source: RCBP of Belém and Ananindeua, 2024.

The incidence of malignant neoplasms was found to be higher in males, with 70.41 cases per 100,000 inhabitants. In turn, for females, it was 66.21 cases per 10,000 inhabitants. It was observed that a large number of cases registered in both sexes occurred in the age group 15–19 years, with 38.5% in females and 35.3% in males (Figure 1).

As for race, most cases occurred in brown patients (57.4%); however, there were a large number of notifications in which this category was marked as ‘no information’ (25.7%). They were followed by white patients (13.5%), black patients (2.8%), indigenous patients (0.5%) and, finally, only one yellow patient, representing 0.1%.

**Figure 2.**
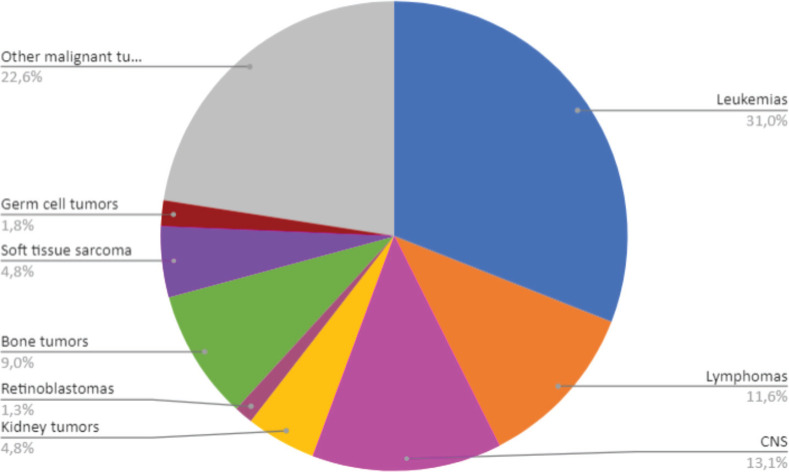
Distribution of malignant neoplasms in Belém and Ananindeua, between 2010 and 2019. Source: RCBP of Belém and Ananindeua, 2024.

In terms of morphology, leukaemia were the most frequent, accounting for 31% of the new cases, followed by malignant CNS tumours, corresponding to 13.1% and with 11.6% of the notifications, there are lymphomas (Figure 2). The other malignant tumours correspond to a total of 22.6% of the cases.

**Figure 3.**
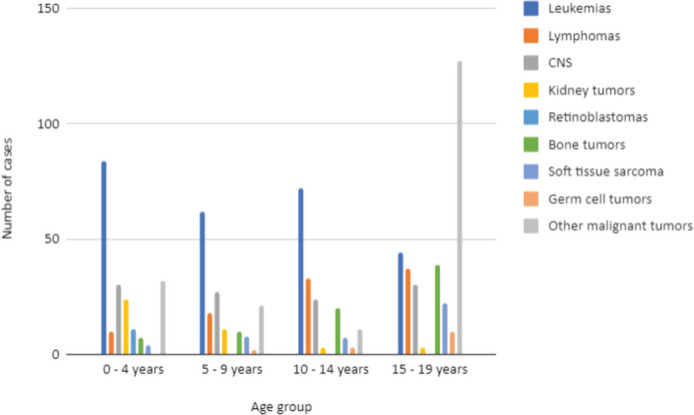
Distribution of malignant neoplasms in Belém and Ananindeua, between 2010 and 2019, according to morphology. Source: RCBP of Belém and Ananindeua, 2024.

Regarding the morphological type according to age group (Figure 3), leukaemias are more frequent among individuals under the age of 15, accounting for 77.42% of notifications in those under 20. In the 15–19 age group, other tumours are more prevalent.

When analysing the distribution of morphological types by age group, malignant neoplasms of the central nervous system (CNS) ranked second in case frequency in the 0–4 years (14.9%) and 5–9 years (17%) groups. In the 10–14 years group (19.1%), lymphomas were the second most frequent (Figure 3).

**Figure 4.**
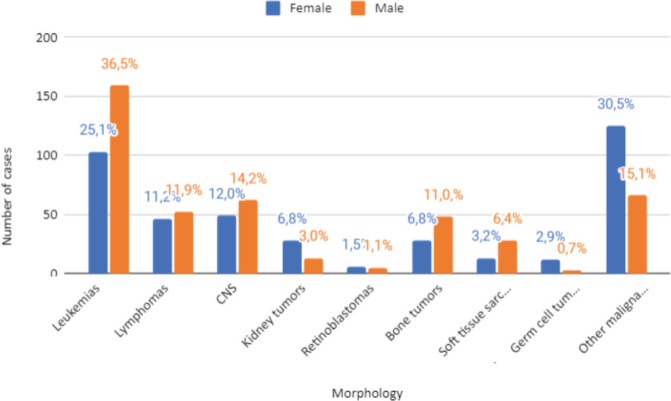
Distribution of malignant neoplasms in Belém and Ananindeua, between 2010 and 2019, according to sex. Source: RCBP of Belém and Ananindeua, 2024.

Regarding morphology by sex (Figure 4), leukaemias remained the most common in both sexes, representing 36.5% of cases in males and 25.1% in females. Less frequent tumours, when combined, accounted for 30.5% and 15.1% in males and females, respectively. These were followed by CNS neoplasms (14.2% in males and 12% in females) and lymphomas (11.9% and 11.2%).

**Figure 5.**
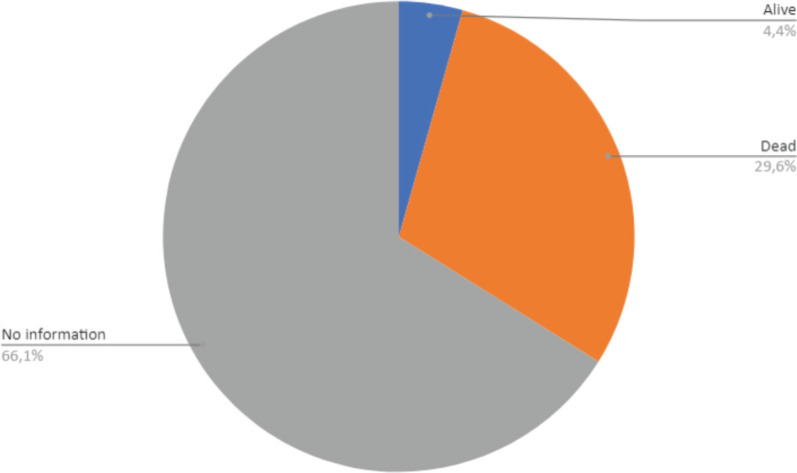
Outcome of malignant neoplasm cases in Belém and Ananindeua, between 2010 and 2019. Source: RCBP of Belém and Ananindeua, 2024.

With regard to the outcome of the new registered cases, 66.1% had no information on the patient’s vital status, and 29.6% of the cases had confirmed death information in the system, but this death may or may not have been related to cancer. Finally, only 4.4% of the cases were registered with a vital status of alive (Figure 5).

## Discussion

The RCBP are reliable sources of information on the incidence of cancer in a given region and are extremely important in cancer epidemiology for implementing public health policies. Using data from the RCBP, it is possible to monitor new cases of malignant neoplasms and their outcomes and, consequently, evaluate the effectiveness of public health actions, as well as formulate new strategies for the prevention of diseases and improvement of the survival of individuals with cancer [14].

Cancer in adults occurs due to genetic changes caused by carcinogens and/or exposures that can lead to both its genesis and progression [1]. Conversely, childhood and adolescent cancer, which occurs in individuals under the age of 20, differs from adult cancer in that its real causes are still poorly understood and there is no need for previous exposure to carcinogenic factors. Nonetheless, it is perceived that this pathology has been showing an increase of approximately 1% per year in its rates worldwide [3, 6].

According to the information collected from the Belém RCBP, it was observed that the majority of the new cases of neoplasms registered in children and adolescents occurred in males, in agreement with another study carried out with data from the RCBP in Goiás, which also showed a higher incidence of malignant neoplasms in this same population [4].

In addition, it was possible to analyse that the age group 15–19 years had the highest number of new cases. These data presented by the Belém RCBP are in line with the trend presented in a study of cancer registries in Latin America and the Caribbean between 2001 and 2010, where it was observed that, among the 36,744 cases of childhood and adolescent cancer, the age-specific incidence rate per million person-years was 132.6 in the age group 0–14, while it was 152.3 in the age group 15–19 [15].

However, the age group 10–14 for females and the age group 5–9 for males had the lowest number of cases. In this context, in Goiás, the incidence rate of cancer in the child and adolescent population is highest in the age groups 0–4 and 15–19 for both sexes [4].

In this study, the brown race had the highest number of new cases. However, in the United States, non-Hispanic white children have a higher incidence of cancer compared to black children [16]; however, the study did not consider the different ethnic groups that can be influenced by ancestral genetic factors in the regions of a country. Thus, the data introduced in this study are in line with the current profile of the

Brazilian population, where, according to the 2022 Demographic Census, 45.3% of Brazilians declare themselves to be brown. In addition, a study using data from Brazil’s Hospital Cancer Registries (RHC, as per its Portuguese acronym) also showed a higher incidence of cancer cases in the brown population [17, 18].

When analysing the morphology of malignant neoplasms, leukaemia were the most frequent in both sexes and in all age groups, with the highest concentration of cases in children aged 0–4 years. The result presented in this research is in line with the study carried out in 2022 with all RHCs in Brazil, which confirms that certain morphologies are more incident at certain ages, and it was observed that leukaemia are the most common type of cancer in children and adolescents, especially in individuals under 5 years of age [17].

Still, in terms of morphology, the second most common type of case corresponded to malignant neoplasms of the CNS, which were more frequent in the age groups from 0–4 and 5–9. In this sense, CNS neoplasms are the most common type of solid tumour in children in the studied period and are one of the main causes of death in children worldwide. In addition, little is known about the etiology of CNS tumours in children, since, unlike cancer in adults, childhood neoplasms do not have environmental and behavioural factors as a relevant cause; however, it is perceived that genetics is the main factor. Furthermore, the diagnosis of this type of neoplasm in childhood is challenging, as the signs and symptoms are non-specific, such as nausea, vomiting, weakness and convulsions, and can be confused with other diseases [19].

Lymphomas follow leukaemia and CNS neoplasms, but the latter is the second most common type in the age group 10–14. A study carried out [20] to estimate the incidence of childhood and adolescent cancer around the world identified leukaemia as the most common type in the population aged 0–15, followed by CNS tumours and lymphomas.

In the age group 15–19, bone tumours are the second most common type of cancer. A survey [21] carried out with data from the US Cancer Epidemiology Surveillance Program, between 2000 and 2019, showed the highest incidence of cases of osteosarcoma in the public consisted of children and adolescents, in the age group 10–19, with an even higher incidence in the male population.

With regard to the outcome of cases of malignant neoplasm, 29.6% of the notifications recorded already had a death outcome at the time of collection, which may or may not be related to cancer. A study [22] on childhood and adolescent cancer mortality in Brazil between 1996 and 2017 showed that, during the analysed period, there were more than 62,000 deaths from malignant neoplasms in Brazil in the age group 0–19. Furthermore, in this study, the age group 0–4 had the highest mortality rates in all regions of Brazil.

However, the study has limitations and, therefore, the 29.6% of the deaths in children with malignant neoplasms cannot be concluded as low mortality in the age group consisting of children and adolescents, since 66.1% of the notifications did not contain any information about this variable. The same is repeated in other variables that had to be removed from the initial project, as 100% of the RCBP notification forms were without information and/or blank in relation to this record, namely: marital status, education, laterality and distant metastasis. All of this puts the quality of the information in this register at risk, but we cannot infer that an RCBP does not have quality information if we only take these variables into account. For this registry, it is recommended that, when making its notifications, due attention should be paid to the information on its variables and an active search should be made for cases that have already been notified, in order to improve the data without information.

## Conclusion

Therefore, this study made it possible to characterise the cases of malignant neoplasms in the child and adolescent population of the Belém and Ananindeua region. Cases were predominant in males and were concentrated in the age group 15–19. In terms of morphology, leukaemia were the most frequent in all age groups, with emphasis on children aged between 0 and 4, registering the highest number of cases.

The need for better records of information on cancer cases in the region should be emphasised due to the high number of ‘no information’ forms in some of the topics investigated in the research. Thus, this study contributes in terms of understanding the epidemiological profile of childhood and adolescent cancer in Belém and Ananindeua, providing important data and information for public health policies for prevention and timely diagnosis in the area of childhood and adolescent oncology.

## Conflicts of interest

The authors declare that they have no conflicts of interest.

## Funding

This research received no external funding.

## Author contributions

IPG and FCRA wrote the manuscript. SMC, GPC, LFS and LACF supervised the whole project. All authors read and approved the final manuscript.

## References

[1] de Oliveira Santos M, Silva de Lima FC, Martins LFL (2023). Estimativa de Incidência de Câncer no Brasil 2023–2025. Rev Bras Cancerol.

[2] World Health Organization (2021). CureAll Framework: WHO Global Initiative for Childhood Cancer. Increasing Access, Advancing Quality, Saving Lives.

[3] Feliciano SVM, Santos MO, Pombo-de-Oliveira MS (2018). Cancer incidence and mortality among children and adolescents: a narrative review. Rev Bras Cancerol.

[4] de Oliveira MM, Silva DRME, Ramos FR (2020). Children and adolescents cancer incidence, mortality and survival a population-based study in Midwest of Brazil. Cancer Epidemiol.

[5] Instituto Nacional de Câncer José Alencar Gomes da Silva (2020). Atlas on-line de mortalidade. https://www.inca.gov.br/MortalidadeWeb/pages/Modelo10/consultar.xhtml#panelResultado.

[6] Algayer LP, Febras LLT, Scheid BS (2020). Tendência Temporal de Internações por Diagnóstico Oncológico em Crianças e Adolescentes. Rev Bras Cancerol.

[7] de Silva VB, Lucena NNN, Pinto RNM (2022). Factors associated with the time between diagnosis and initiation of childhood cancer treatment. Rev Saude Pesquisa.

[8] Mutti CF, Cruz VG, Santos LF (2018). Clinical and epidemiological profile of children and adolescents with cancer in an oncology service. Rev Bras Cancerol.

[9] Marinho CSR, Flor TBM, Pinheiro JMF (2020). Objetivos de Desenvolvimento do Milênio: impacto de ações assistenciais e mudanças socioeconômicas e sanitárias na mortalidade de crianças. Cad Saúde Pública.

[10] Instituto Nacional de Câncer (2022). Registros de câncer de base populacional. https://www.inca.gov.br/numeros-de-cancer/registro-de-cancer-de-base-populacional.

[11] Secretaria de Estado de Saúde Pública do Pará (2010). Retrato do câncer infantil em Belém e Ananindeua (1999–2001).

[12] Latorre MRDO, Almeida ABM, Möller BB (2021). A Importância do registro de câncer no planejamento em saúde. Rev USP.

[13] Instituto Brasileiro de Geografia e Estatística (IBGE) (2012). Censo Brasileiro de 2010.

[14] Gil F, Vries E, Wiesner C (2019). Importancia del acceso de los registros de cáncer de base poblacional a las estadísticas vitales: barreras identificadas en Colombia. Rev Colomb Cancerol.

[15] de Paula Silva N, Colombet M, Moreno F (2024). Incidence of childhood cancer in Latin America and the Caribbean: coverage, patterns, and time trends. Rev Panam Salud Publica.

[16] Lupo PJ, Spector LG (2020). Cancer progress and priorities: childhood cancer. Cancer Epidemiol Biomarkers Prev.

[17] de Lucena NNN, Damascena CLL, Moreira MSC (2022). Characterization of childhood cancer in Brazil from the hospital-based cancer registries, 2000–2016. Rev Pesqui Cuidado Fundam Online.

[18] Instituto Brasileiro de Geografia e Estatística (IBGE) (2023). Censo Demográfico 2022.

[19] Magalhães GA, Magalhães DM, Bellas GO (2023). Análise Epidemiológica, Clínica e Patológica de Crianças com Neoplasias do Sistema Nervoso Central Tratadas com Radioterapia no Instituto Nacional de Câncer. Rev Bras Cancerol.

[20] Johnston WT, Erdmann F, Newton R (2021). Childhood cancer: estimating regional and global incidence. Cancer Epidemiol.

[21] Liu T, Cui L, He Z (2023). Epidemiology and nomogram of pediatric and young adulthood osteosarcoma patients with synchronous lung metastasis: a SEER analysis. PLoS One.

[22] Velame KT, Antunes JLF (2024). Cancer mortality in childhood and adolescence: analysis of trends and spatial distribution in the 133 intermediate Brazilian regions grouped by macroregions. Rev Bras Epidemiol.

